# Epigenetic-related gene mutations serve as potential biomarkers for immune checkpoint inhibitors in microsatellite-stable colorectal cancer

**DOI:** 10.3389/fimmu.2022.1039631

**Published:** 2022-11-21

**Authors:** Chao Liu, Huiting Xiao, Luying Cui, Lin Fang, Shuling Han, Yuli Ruan, Wenyuan Zhao, Yanqiao Zhang

**Affiliations:** ^1^ Department of Gastrointestinal Medical Oncology, Harbin Medical University Cancer Hospital, Harbin, China; ^2^ Clinical Research Center for Colorectal Cancer in Heilongjiang, Harbin Medical University Cancer Hospital, Harbin, China; ^3^ Key Laboratory of Tumor Immunology in Heilongjiang, Harbin Medical University Cancer Hospital, Harbin, China; ^4^ College of Bioinformatics Science and Technology, Harbin Medical University, Harbin, China; ^5^ Translational Medicine Research and Cooperation Center of Northern China, Heilongjiang Academy of Medical Sciences, Harbin, China

**Keywords:** immune checkpoint inhibitor therapy, biomarker, microsatellite-stable, colorectal cancer, epigenetic-related gene mutations

## Abstract

**Background:**

Combination therapy with immune checkpoint inhibitors (ICIs) may benefit approximately 10-20% of microsatellite-stable colorectal cancer (MSS-CRC) patients. However, there is a lack of optimal biomarkers. This study aims to understand the predictive value of epigenetic-related gene mutations in ICIs therapy in MSS-CRC patients.

**Methods:**

We analyzed DNA sequences and gene expression profiles from The Cancer Genome Atlas (TCGA) to examine their immunological features. The Harbin Medical University Cancer Hospital (HMUCH) clinical cohort of MSS-CRC patients was used to validate the efficacy of ICIs in patients with epigenetic-related gene mutations (Epigenetic_Mut).

**Results:**

In TCGA, 18.35% of MSS-CRC patients (78/425) had epigenetic-related gene mutations. The Epigenetic_Mut group had a higher tumor mutation burden (TMB) and frameshift mutation (FS_mut) rates. In all MSS-CRC samples, Epigenetic_Mut was elevated in the immune subtype (CMS1) and had a strong correlation with immunological features. Epigenetic_Mut was also associated with favorable clinical outcomes in MSS-CRC patients receiving anti-PD-1-based therapy from the HMUCH cohort. Using immunohistochemistry and flow cytometry, we demonstrated that Epigenetic_Mut samples were associated with increased anti-tumor immune cells both in tumor tissues and peripheral blood.

**Conclusion:**

MSS-CRC patients with epigenetic regulation impairment exhibit an immunologically active environment and may be more susceptible to treatment strategies based on ICIs.

## Introduction

Immune checkpoint inhibitor (ICI) therapy has achieved impressive success in deficient mismatch repair (dMMR)/microsatellite instability-high (MSI-H) colorectal cancer (CRC). ICI has been considered a standard therapy by the FDA, including the use of programmed death receptor 1 (PD-1) monoclonal antibodies and CTL-associated protein 4 (CTLA-4) monoclonal antibodies (1–3). However, the vast majority of CRC cases (approximately 85%) are characterized by proficient mismatch repair (pMMR)/microsatellite stability (MSS) tumors that do not respond to ICIs (4). Recent studies suggest that a subgroup (approximately 10-20%) of MSS-CRC patients might benefit from combination regimens of ICIs (5–7). Therefore, predictive biomarkers for screening these patients are urgently needed.

Current clinical and investigational studies of screening MSS-CRC patients who would benefit from ICIs treatment are limited. PD-L1 expression is a classic biomarker, but the Keynote-028 study demonstrated that PD-L1^+^ MSS-CRC patients could not benefit from ICI therapy (8). *POLD1*/*POLE* mutations are predictive but occur in only 1% of MSS-CRC patients (9). Biomarkers such as tumor mutation burden (TMB), tumor-infiltrating lymphocytes (TIL), neo-antigen load (NAL), and immune-regulatory gene expression profiling (iGEP) may allow the selection of clinical patients for ICIs. However, the lack of uniform detection methods and validated cutoffs limit the use of these methods (5, 10–12). Several emerging biomarkers, such as gut microbiota and T-cell-receptor (TCR) sequencing, have also shown predictive value, although they are not yet clinically applicable (7, 13). DNA damage response (DDR) gene mutations may induce a hypermutational phenotype (14), and recent studies have shown that patients with MSS-CRC and mutations in the DDR system have better immune responses and outcomes following ICI therapy (15, 16). However, the pathogenicity of different DDR gene mutations in MSS-CRC remains unclear, and their incidence is significantly lower than in endometrial, ovarian, or biliary tract cancers (17).

Epigenomic alterations can affect tumor immunogenicity and anti-tumor responses by regulating genome stability and chromatin accessibility (18). Additionally, several epigenetic-related gene mutations have been shown to exhibit predictive functions in ICI therapy for multiple types of tumors. ARID1A, an AT-rich interactive domain-containing protein 1A, is a component of the switching defective/sucrose non-fermenting (SWI/SNF) complex that plays a role in chromatin remodeling (19), and increasing evidence suggests that ARID1A alterations are correlated with better outcomes after ICI therapy for bladder cancer, nonsmall-cell lung cancer (NSCLC), and gastric cancer (20, 21). ARID1A mutation is defined as an immunologically active subgroup in MSS-CRC patients with abundant intra-tumoral T-cell infiltration (22). Lysine methyltransferase 2 (KMT2) family members facilitate transcription and gene accessibility by methylating lysine 4 on histone H3 (H3k4) (23), and *KMT2* family mutations have also been linked to a favorable response to ICIs in multiple cancers (24). Furthermore, as identified using clustered regularly interspaced short palindromic repeats (CRISPR), KMT2D mutant tumors exhibit an increased mutation burden, IFN-γ-stimulated antigen presentation, and a higher sensitivity to ICIs. Moreover, disruption of DNA methylation signatures has been identified as a marker of anti-PD-1 therapy efficacy in NSCLC (25), and TET1, a DNA demethylase, enhances the immunotherapeutic effect (26). Although this evidence points to the role of epigenetic regulation in anti-tumor immune responses, there is no clinical data on the association between comprehensive epigenetic-related gene mutations (mutations in genes that are involved in epigenetic modifications) and the clinical benefit of ICIs in MSS-CRC.

Given the proposed role of epigenetic regulation impairment in predicting the response to ICIs, we hypothesize that epigenetic-related gene mutations in MSS-CRC cause hypermutation and improve the expression of immune response gene sets. As a result, we conducted this study to clarify the value of epigenetic-related gene mutations as an indicator of immunotherapy efficacy in patients with MSS-CRC. For this purpose, we analyzed whole-exome sequencing (WES) data from TCGA to study TMB, frameshift mutation (FS-mutation), and immune characteristics of Epigenetic_Mut and Epigenetic_Wt groups of MSS-CRC samples. Additionally, in a Chinese clinical MSS-CRC cohort of 89 patients who received PD-1-based treatment, we found that Epigenetic_Mut was associated with favorable clinical outcomes. Here, we report the relationships between epigenetic-related gene mutations and TMB, FS-mutation, immunomodulatory mRNA expression signature, and ICI therapy efficacy in patients with MSS-CRC.

## Materials and methods

### Patient information and sample collection

To determines the incidence of epigenetic-related gene mutations in MSS-CRC, we analyzed DNA sequencing and gene expression profiles of 514 MSS-CRC patients from two cohorts: (1) a TCGA cohort consisting of 425 MSS-CRC patients and (2) a HMUCH cohort comprising 89 Chinese patients with annotated response and mutational data from Harbin Medical University Cancer Hospital (the inclusion and exclusion criteria are shown in **Supplementary Figure 1**). This study was approved by the Ethics Committee of the Harbin Medical University Cancer Hospital (No. KY2022-20).

### Epigenetic-related gene status definition

Epigenetic-related gene status (Epigenetic_Wt or Epigenetic_Mut) was defined based on the presence of a loss-of-function (LOF) variant in 68 genes that have been proposed as core genes of epigenetic regulation (18). **Supplementary Table 1** presents a detailed description. Nonsense, frameshift, and splice site changes within consensus regions and start lost/gained variants were considered to be LOF variants. Missense and in-frame variants were excluded from the analysis.

### DNA extraction and sequencing

For the TCGA cohort, gene mutation data were acquired using the GDC Data Portal. We assessed the mutational status of epigenetic-related genes in CRC using exome-sequencing data from HMUCH. For analysis, DNA was extracted using a DNA Kit (Applied Biosystems, Foster City, CA, USA), from whole blood samples or formalin-fixed paraffin-embedded (FFPE) tissues of each patient. The lymphocytes from the whole blood samples were isolated by centrifugation at 1,600 × g for 10 min in red cell lysis buffer (Tiangen, RT122, Beijing, China) at 25°C, and DNA was extracted using a genomic DNA kit (Tiangen, DP304, Beijing, China). We sheared the DNA into fragments of 150-200 bp using an ultrasonicator and used a KAPA Kit (KAPA Biosystems, Wilmington, MA, USA) to prepare DNA fragment libraries for the Illumina platform (Illumina HiSeq X-Ten, Illumina, USA). Probe hybridization capture technology and Illumina high-throughput sequencing were used to detect the exonic regions and some intronic regions of 825 tumor-related genes (Genetron Health Co., Ltd. Beijing, China) (**Supplementary Table 2**).

### Analysis of MSI status, TMB, and FS-mutation in the TCGA and HMUCH cohorts

MSI status for the TCGA cohort was determined using the MSI sensor (version 0.5). In brief, for MSI sensor scores < 3.5, samples were considered to be MSS; otherwise, they were considered MSI (27). Published studies using the TCGA cohort provided FS-mutation and TMB data (28–30), and MSI status for the HMUCH cohort was determined using a 3730 sequencer (Life Technologies, Carlsbad, CA, USA). For this purpose, whole blood samples or prepared FFPE tissue were diluted to 2 ng/μL or 20 ng/μL, respectively, followed by the addition of 2.8 μL of ddH_2_O, 4 μL of 2.5× Buffer A, 2 μL of 5× MSI Primer Mix, and 0.2 μL of Taq DNA Polymerase I. PCR amplification was carried out as follows: pre-denaturation at 95°C for 5 min; followed by 30 cycles at 94°C for 30 s, 60°C for 1 min, and 70°C for 1 min; and then a final extension at 60°C for 30 min. Finally, the temperature was reduced to 15°C, and the samples were centrifuged at 3,000 × g for 1 min. NR-21 and BAT-26 were labeled with blue fluorescent dye, BAT-25 with green dye, and NR-24 and MONO-27 with yellow dye. Finally, tumors were classified as MSI-H if two or more markers showed instability; otherwise they were classified as MSS.

### Analysis of the consensus molecular subtypes (CMSs) in the TCGA cohort

Consensus molecular subtypes (CMSs) are classification systems for CRC and include immune (CMS1), canonical (CMS2), metabolic (CMS3), and mesenchymal (CMS4) subtypes. These subtypes were identified through a large-scale analytical study and have unique molecular and metabolic characteristics (31).

### Immune-related signature analysis

Our study compared the RNA expression of patients with Epigenetic_Mut and Epigenetic_Wt using gene signatures for the IFN-γ pathway and other immunological responses (**Supplementary Table 3**) (12, 32). We obtained TCGA transcriptome profiles from the GDC data portal, and used transcripts per kilobase million (TPM) normalization to normalize gene expression. The geometric mean of gene expression levels in the log_2_ (TPM + 1) format was used to evaluate immune signatures.

### Clinical outcomes

The objective response rate (ORR), disease control rate (DCR), progression-free survival (PFS), and overall survival (OS) were the main clinical outcomes of interest. The Response Evaluation Criteria in Solid Tumors (RECIST) version 1.1 was used for the assessment of ORR and divided into complete response (CR) and partial response (PR). DCR was defined as CR, PR, or stable disease (SD) lasting more than six months. PFS was evaluated from when immunological therapy was initiated until progression or death, and patients who did not progress were examined at the last scan. OS was evaluated from the start of ICI therapy until patient death or the end of the trial, and the patients with whom we lost contact were classified based on the date of last contact.

### Immunohistochemistry (IHC)

Primary tumor paraffin sections of 4 μm were processed for immunochemistry to evaluate CD8^+^ and FOXP3^+^ lymphocytes according to the following protocol: roast, deparaffination, and rehydration before performing heat-mediated antigen retrieval with EDTA buffer (pH 9.0), inactivation of endogenous peroxidase activity with 3% H_2_O_2_, incubation with antibody against CD8 (ab101500, 1:500; Abcam, Cambridge, UK) or against FOXP3 (ab200334, 1:500; Abcam) at 4°C overnight, exposure to a DAB IHC Detection Kit after incubation with biotinylated secondary antibodies, and counterstaining with Mayer’s hematoxylin solution. An open-source platform for biological-image analysis (Fiji/ImageJ) was used to estimate the densities of CD8^+^ and FOXP3^+^ lymphocytes.

### Flow cytometry analysis

The peripheral blood mononuclear cells (PBMC) of CRC patients were isolated by centrifugation with erythrocytes lysate and were used to analyze PD1^+^CD8^+^T cells and CD3^-^CD56^+^CD16^+^NK cells by flow cytometry. The PBMC were stained for 30min on ice using the following antibodies: FITC anti-human CD8 (344704, Biolegend), PE anti-human PD1(367404, Biolegend), APC anti-human CD3 (300312, Biolegend), PE anti-human CD56 (985902, Biolegend), and PerCP anti-human CD16 (302030, Biolegend). Stained cell suspensions were analyzed using the BD flow cytometer (BD Accuri C6 Plus), and data analysis was performed using FlowJo_v10.8.1.

### Statistical analysis

Fisher’s exact test was used to analyze the relationship between epigenetic-related gene mutations and the ORR or DCR, and the Kaplan–Meier method and log-rank test were employed to examine the PFS and OS probabilities of the Epigenetic_Mut and Epigenetic_Wt CRC groups. Based on the Mann–Whitney U-test, TMB, FS-mutation, tumor-infiltrating lymphocytes, expression of immune-related genes, and immune signatures were compared between the Epigenetic_Mut and Epigenetic_Wt CRC groups. Statistical analysis was conducted using two-sided tests with a nominal significance level of 0.05 using R version 3.5.2.

## Results

### The mutational landscape of epigenetic-related genes of MSS-CRC in the TCGA cohort

**Figure 1:**
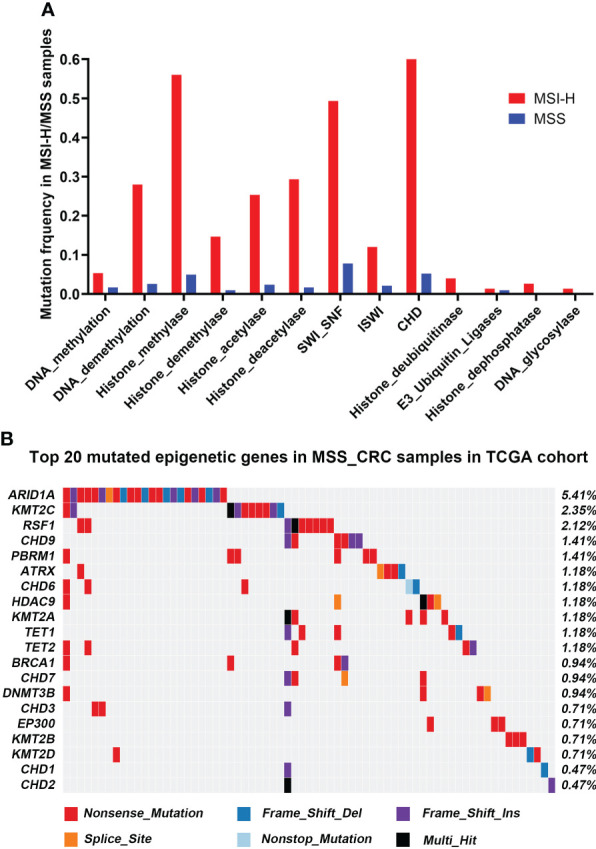
Mutational landscape of Epigenetic-related genes associated with MSS-CRC cases from the TCGA and HMUCH cohorts. **(A)** The frequency of epigenetic regulatory pathway alteration in MSS-CRC cases and MSI_H samples from the TCGA cohort. **(B)** The top 20 mutated epigenetic-related genes in MSS-CRC samples from the TCGA cohort.

A total of 68 epigenetic-related genes involved in 13 different pathways were included in the current research, including genes involved in modifying DNA, histones, and protein complexes that reshape chromatin structure (**Supplementary Table 1**). In the TCGA cohort, MSI-H and MSS-CRCs had epigenetic-related gene mutation frequencies of 66.67% (50/75) and 18.35% (78/425), respectively. The three most frequently mutated pathways in the MSS-CRC cases from TCGA were SWI_SNF, Histone_methylase, and CHD (**Figure 1A**), and the epigenetic-related genes ARID1A, KMT2C, and RSF1 had the highest mutation rates in the TCGA cohort (**Figure 1B**).

### Epigenetic-related gene mutations are linked with the TMB, FS-mutation, and molecular subtype of CRC

**Figure 2:**
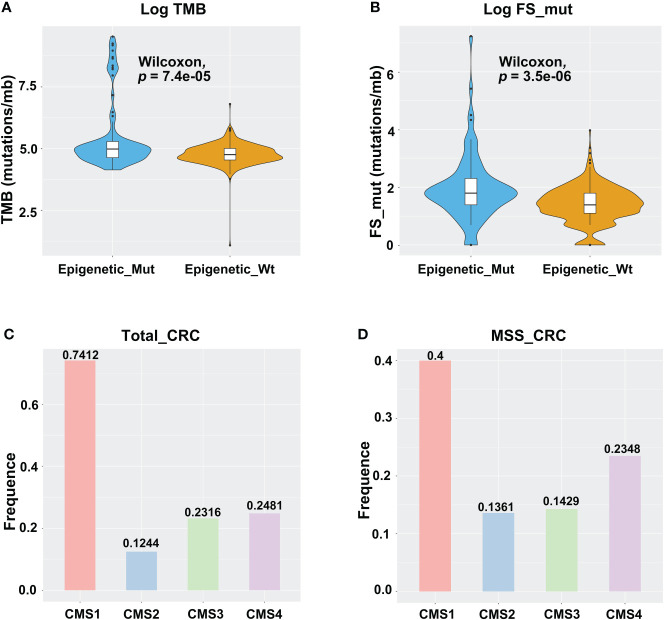
Epigenetic-related gene mutations are linked with the TMB, FS-mutation, and molecular subtypes of CRC. **(A)** TMB violin plot of Epigenetic_Mut and Epigenetic_Wt from MSSCRC samples. **(B)** FS-mutation rate violin plot of Epigenetic_Mut and Epigenetic_Wt from MSS-CRC samples. **(C)** Molecular subtype-specific fold enrichment of epigenetic-related genes mutation in all CRC cases (MSI-H/MSS). **(D)** Molecular subtype-specific fold enrichment of ARID1A mutation in MSS-CRC.

High levels of TMB and FS-mutations (FS_mut) reflect a high degree of genomic instability and potential immunogenicity of a tumor, and both of these are therefore potential biomarkers of immune checkpoint inhibitor responsiveness. Hence, we examined the relationships between TMB, FS_mut, and epigenetic-related gene mutation status. In TCGA cohort, epigenetic-related gene mutations were associated with an increased incidence of TMB in MSS-CRC (median mutation rate of 4.76/mb vs. 4.99/mb in Wt and Mut cases, respectively; *p* = 7.4e-05; **Figure 2A**). A higher rate of FS_mut was also linked with epigenetic-related gene mutations in MSS-CRC (median frameshift mutation rate of 1.39/mb vs. 1.79/mb in Wt and Mut cases, respectively, *p* = 3.5e-06; **Figure 2B**). Molecular subtypes of CRC (CMS) are currently a highly recognized classification method for CRC that can accurately guide patient treatment and prognosis. CMS1, also known as the immune subtype, has better immune activity and high reactivity to ICIs. Here, we analyzed the distribution of Epigenetic_Mut samples based on molecular subtype in all CRC and MSS-CRC cases (**Figures 2C, D**). Among the CMS1-CRC cases, 74.12% were Epigenetic_Mut samples (74.12%, 63/85), but in MSS CMS1-CRC cases, this rate was 40% (12/30). Both for all samples and MSS CRC specifically, Epigenetic_Mut samples were enriched in the CMS1 (immune subtype) group.

### Epigenetic_Mut is related to increased immune activity in MSS CRC

**Figure 3:**
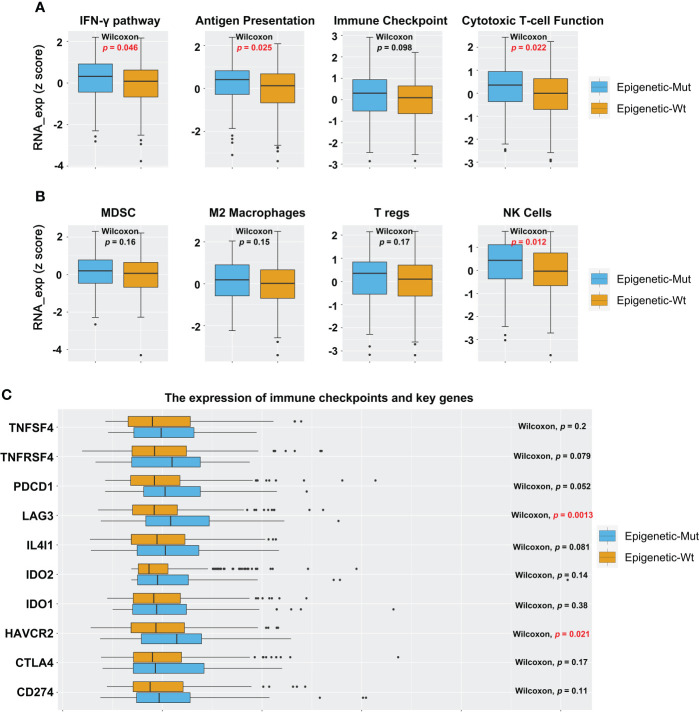
Epigenetic_Mut is associated with increased immune activity in MSS-CRC. **(A)** The RNA expression of immune response gene sets in MSS-CRC based on the epigenetic-related genes’ mutational status. **(B)** The RNA expression of immune cells gene sets in MSS-CRC based on the epigenetic-related genes’ mutational status. **(C)** The RNA expression of a single immune response gene in MSS-CRC based on the epigenetic-related genes’ mutational status.

To identify the tumor immune microenvironment, we compared Epigenetic_Mut and Epigenetic_Wt for immune signatures, tumor-infiltrating lymphocytes, and expression of immune checkpoints and key genes. We demonstrated that epigenetic-related gene mutations increased the expression of immune response genes, including those involved in the IFN-γ pathway, antigen presentation, and cytotoxic T-cell function (**Figure 3A**). In addition, the expression of NK cell-related genes was increased in the Epigenetic_Mut group. Other immune cells also showed an upward trend, but no statistical difference was observed due to the limited cohort size (**Figure 3B**). Finally, we compared the expression of immune checkpoints and key genes between the two groups. In line with the immune response pathway, several immune checkpoints and key genes were upregulated in the Epigenetic_Mut group. In particular, the expression of *LAG3* and HAVCR2 was significantly elevated, and elevated levels of *TNFRSF4*, *PDCD1*, and *IL4l1* were very nearly statistically significant (**Figure 3C**).

### Epigenetic_Mut predicts favorable clinical outcomes following ICI therapy

**Figure 4:**
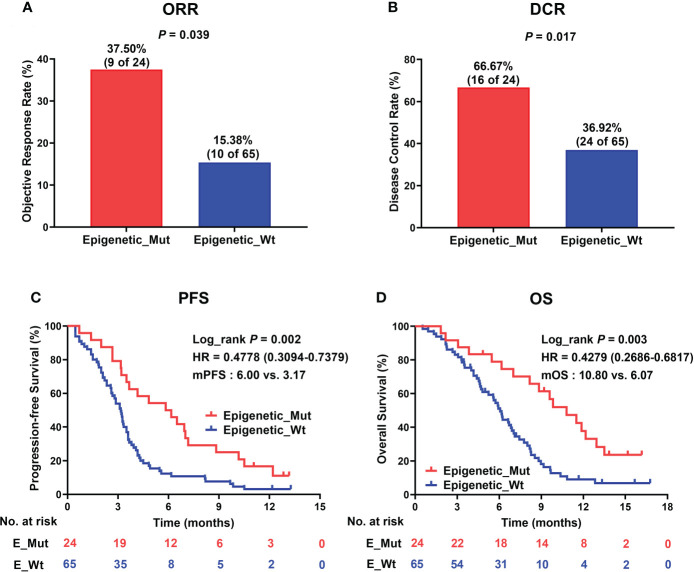
Epigenetic_Mut predicts favorable clinical outcomes following ICI therapy. **(A)** Histogram presenting the proportion of patients that acquired ORR in the Epigenetic_Mut and Epigenetic_Wt groups. **(B)** Histogram presenting the proportion of patients that acquired DCR in the Epigenetic_Mut and Epigenetic_Wt groups. **(C)** Kaplan–Meier estimates of PFS between Epigenetic_Mut or Epigenetic_Wt group patients in the discovery cohort. **(D)** Kaplan–Meier estimates of OS between Epigenetic_Mut or Epigenetic_Wt group patients in the discovery cohort.

Next, to validate the function of epigenetic-related gene mutations further in predicting responsiveness to ICI therapy in MSS-CRC, we collected a clinical cohort of 89 MSS-CRC patients who had received PD-1 mAb-based treatment. **Table 1** shows the baseline patient characteristics based on epigenetic-related gene status. Of the 89 patients, 24 had Epigenetic_Mut, and 65 had Epigenetic_Wt. Using RECIST version 1.1, we evaluated the patients’ best overall responses. Compared to Epigenetic_Wt, Epigenetic_Mut had a significantly higher ORR (**Figure 4A**, 37.50% (9/24) vs. 15.38% (10/65), Fisher’s exact test *P* = 0.039). As for DCR, the rate was 66.67% (16/24) in patients with epigenetic-related gene mutations from ICI treatment but only 36.92% (24/65) in patients without epigenetic-related gene mutations (**Figure 4B**, Fisher’s exact test *P* = 0.017). As expected, PFS was greatly improved in patients with epigenetic-related gene mutations compared to those without epigenetic-related gene mutations in this cohort (**Figure 4C**, mPFS:6.00 vs. 3.17 months, Log_rank *P* = 0.002, HR = 0.4778), and ICI treatment also had a greater benefit on OS in the Epigenetic-Mut group than that in the Epigenetic-Wt group. (**Figure 4D**, mOS: 10.80 vs. 6.07 months, Log_rank *P* = 0.003, HR = 0.4279). In addition, we screened 9 genes with high mutation frequency from all epigenetic-related genes, whose predictive value has been demonstrated in other solid tumors, including ARID1A, ATRX, KMT2A/B/C/D, and TET1/2/3. The results showed that MSS-CRC with these gene mutations had more considerable ORR (**Supplementary Table 4**, 8/16, 50%) and DCR (**Supplementary Table 4**, 13/16, 81.25%).

**Table 1:** Patient and disease characteristics of the validation set of MSS-CRC patients receiving ICI therapy.

Characteristics	Epigenetic_Mut(n = 24)	Epigenetic_Wt (n = 65)	P-value*
**Age**			0.651
<60	12	36	
≥60	12	29	
**Sex**			0.423
Male	13	36	
Female	11	29	
**ECOG PS**			0.857
0	16	42	
≥1	6	23	
**ICI line**			0.967
1	0	2	
2	6	16	
≥3	18	47	
**Primary tumor sidedness**			0.334
Right	10	20	
Left	14	45	
**Liver metastases**			0.683
With Liver	10	24	
Without Liver	14	41	
**Regimen**			0.951
ICIs + TKIs	10	26	
ICIs + Chemotherapy	11	32	
ICIs + Chemoradiotherapy	3	7	
Best overall response**			0.026
CR/PR	9	10	
SD	7	14	
PD	8	41	

* Fisher’s exact test or Wilcoxon-Mann-Whitney test, as appropriate.

** CR, complete response; PR, partial response; SD, stable disease; PD, progressive disease.

### The abundance of immune cells in tumor tissue and peripheral blood of patients with or without epigenetic-related gene mutation

**Figure 5:**
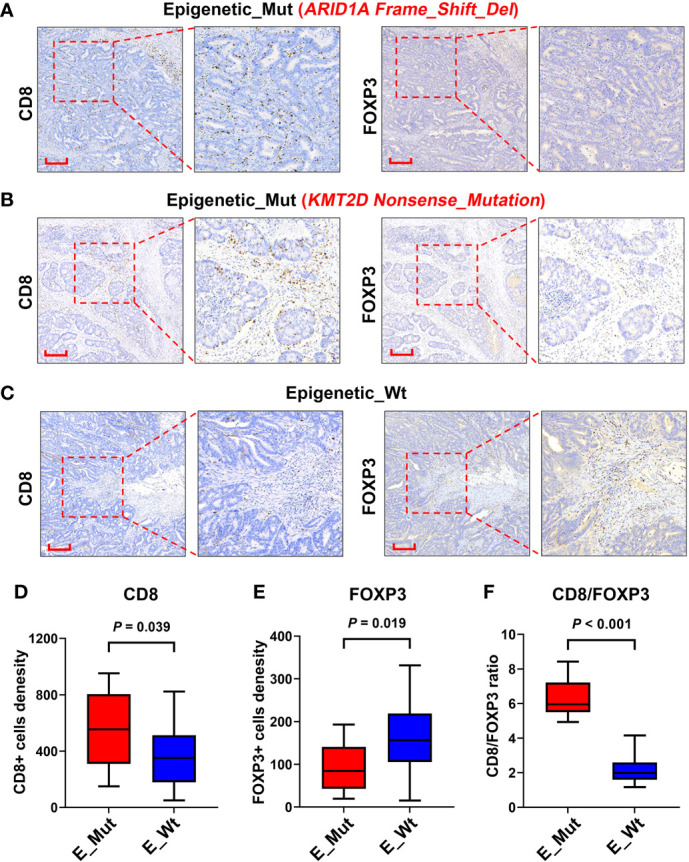
Infiltration of CD8^+^ and FOXP3^+^ lymphocytes in the tumors of patients with or without epigenetic-related gene mutations. **(A)** A Representative image of CD8^+^ and FOXP3^+^ lymphocytes infiltrating the MSS-CRC with ARID1A Frame_Shift_Del. **(B)** A Representative image of CD8^+^ and FOXP3^+^ lymphocytes infiltrating the MSS-CRC with KMT2D Nonsense_Mutation. **(C)** A Representative image of CD8^+^ and FOXP3^+^ lymphocytes infiltrating the MSS-CRC without epigenetic-related gene mutations. **(D)** Tumors with epigenetic-related genes mutation had significantly higher levels of intra-tumoral CD8^+^ lymphocytes than tumors with wild-type epigenetic-related genes. **(E)** Tumors with epigenetic-related genes mutation had significantly lower levels of intra-tumoral FOXP3^+^ lymphocytes than tumors with wild-type epigenetic-related genes. **(F)** The Epigenetic_Mut group had a higher CD8/FOXP3 cell ratio than the Epigenetic_Wt group.

**Figure 6:**
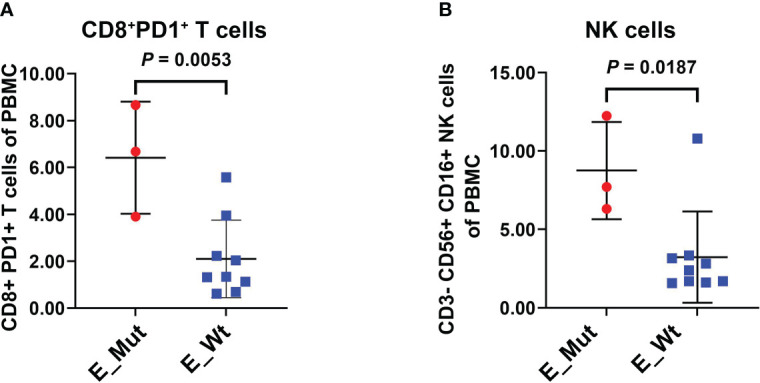
Proportion of CD8^+^PD1+T cells and NK cells in the peripheral blood of patients with or without epigenetic-related gene mutations. **(A)** The Epigenetic_Mut group had a higher proportion of CD8^+^PD1^+^T cells compared to the Epigenetic_Wt group in peripheral blood. **(B)** The Epigenetic_Mut group had a higher proportion of NK cells compared to Epigenetic_Wt group in peripheral blood.

We explored the densities of CD8^+^ and FOXP3^+^ cells in MSS-CRC samples with different epigenetic-related gene statuses using IHC. Of the 34 MSS-CRC samples, 10 had epigenetic gene mutations. Further, we captured representative images of CD8^+^ cells and FOXP3^+^ cells from three samples. The first patient had an *ARID1A* mutation (*ARID1A Frame_Shift_Del*), and the second patient had a *KMT2D* mutation (*KMT2D Nonsense_mutation*). Both samples showed increased CD8^+^ cell density and decreased FOXP3^+^ cell density in tumor tissues (**Figures 5A, B**). However, in the third patient, who did not present any epigenetic-related gene mutations, the density of CD8^+^ lymphocytes was lower, and the density of FOXP3^+^ lymphocytes was higher than that of the other two (**Figure 5C**). CD8^+^ and FOXP3^+^ cell densities were counted in 38 patients (Epigenetic_Mut, N = 10; Epigenetic_Wt, N = 28), and from this we discovered that CD8^+^ cell density increased in the Epigenetic_Mut group (**Figure 5D**) and that the FOXP3^+^ cell density decreased in the Epigenetic_Mut group (**Figure 5E**). Furthermore, the ratio of CD8/FOXP3 cells in the Epigenetic_Mut group was significantly higher than that in the Epigenetic_Wt group (**Figure 5F**). Next, we collected peripheral blood from 12 patients with MSS-CRC (3 with epigenetic-related gene mutations) and measured the proportion of CD8^+^PD1^+^T cells and CD3^-^CD56^+^CD16^+^NK cells by flow cytometry. We found that both the proportion of CD8^+^PD1^+^T cells and CD3^-^CD56^+^CD16^+^NK cells was higher in the Epigenetic_Mut group (**Figures 6A, B**).

## Discussion

Although ICI-based combination therapies have shown certain effectiveness in pMMR/MSS CRC, especially in combination with antiangiogenic agents (Lenvatinib or Regorafenib) that resulted in an ORR of 20-30% (6, 33), most patients still cannot benefit from the combination therapy because of the high heterogeneity of pMMR/MSS CRC. Recently, the MAYA phase II trial (NCT03832621) showed that MSS-CRC patients with silenced MGMT could benefit from ICIs combined with temozolomide treatment (34). This trial showed 36% for 8-month PFS, 42% for ORR, and 18.4 months for the median OS. Therefore, screening the MSS CRC patients with active anti-tumor immune response may be the key to improving the efficacy of immunotherapy. However, the predictive biomarkers for ICI therapy in MSS-CRC patients are limited.

pMMR/MSS CRC is a cold tumor that contains few neoantigens and either no or inactive TILs (35). Meanwhile, CRC is a multilayered heterogeneous disease with specific treatment challenges and opportunities (36). Additionally, Previous studies have reported that epigenetic-related gene mutations affect both the tumor microenvironment and efficacy of ICIs (24–26). Mechanistically, epigenetic modification can reshape the tumor microenvironment by affecting genomic instability and enhancing the immunogenicity of tumor cells. First, epigenetic modification can affect the DNA damage repair response by regulating the accessibility of chromatin. Studies have shown that epigenetic-related gene mutations can lead to increased TMB in tumor cells, such as ARID1A and KMT2D. ARID1A specifically has a 6.7% mutation rate in MSS-CRC (22) and may increase the instability of the genome by adjusting the MMR pathway (21, 37). Mutations in the KMT2D gene are common in cancer patients, and their deficiency can increase the levels of genomic DNA damage and TMB, as well as increase transcription instability. Clinical studies have shown that individuals with mutations in genes from the KMT family are more likely to benefit from ICI therapy (24, 25). Furthermore, epigenetic-related gene mutation enhances the immunogenicity of tumor cells. Accounting for 5%-10% of genomic DNA sequences, human endogenous retroviruses (ERVs) are remnants of the evolution of germline integrations of exogenous infectious retroviruses (38, 39). These exogenous genes are not expressed in healthy tissues other than germ cells but are often abnormally expressed in tumors with epigenetic regulation defects. Here, neoantigen expression increases immunogenicity and triggers an innate immune response against tumors (40, 41). Recently, genome-wide technologies have revealed frequent mutations in epigenetic modifier genes, particularly in cancers (42). It is therefore necessary to analyze systematically the immune activity and the effect of immunotherapy in MSS-CRC patients with epigenetic regulation impairment.

In our study, we systematically analyzed 68 epigenetic-related genes from 13 pathways involved in chromatin regulatory processes in MSS-CRC samples. The mutation rate of epigenetic-related genes in the TCGA cohort was 18.35%. This mutation frequency was higher than that of any previous marker in the population, such as *POLE* or DDR mutations, and was closer to the potential benefit ratio in MSS-CRC clinical trials. *ARID1A*, *KMT2C*, *RSF1*, *CHD9, PBRM1*, and *ATRX* were the most mutated genes in the TCGA cohort, accounting for approximately 75% of the epigenetic-related gene-mutated MSS-CRC patients. This is consistent with previous reports, and *ARID1A* is thus a marker gene that should be investigated in clinical practice.

Using bioinformatics algorithms, we also assessed whether the MSS-CRC samples with epigenetic-related gene mutations from TCGA had better immune activity, including immune signatures, tumor-infiltrating lymphocytes, and expression of immune checkpoints and key genes. Furthermore, we validated our bioinformatic findings using immunohistochemical analyses of CD8^+^ and FOXP3^+^ cells from a cohort of MSS-CRC patients, and similar results were obtained at the histopathological level. In the Epigenetic _Mut group, CD8^+^ cells were higher and FOXP3^+^ cells were lower. The Epigenetic _Mut group also had a higher proportion of CD8/FOXP3 cells than the Epigenetic_Wt group. The VOLTAGE trial demonstrated that among MSS-CRC patients receiving ICIs as neoadjuvant treatment, patients with an elevated CD8/FOXP3 cell ratio were more likely to achieve pathologic complete response (pCR), suggesting that the CD8/FOXP3 cell ratio may be a predictor for ICI therapy efficacy (43).

Finally, we validated the predictive power of epigenetic-related gene mutations in the HMUCH cohort of 89 MSS CRC patients who received immunotherapy and discovered that patients with epigenetic mutations were more likely to benefit from ICI-based combination therapy and had better clinical outcomes. These preliminary results demonstrate that epigenetic-related gene mutations can predict the response to ICIs in MSS-CRC patients.

This study has several limitations, including the validation cohort coming from a single-center, the small size of the cohort, and the lack of validation in other populations. This is because ICI-based regimens have not been recommended by any clinical guidelines for MSS-CRC. Numerous patients included in this study experienced the failure of standard treatment, and the treatment compliance and completeness of the clinical information in many of these patients, were not ideal. Additionally, since the genetic information in the HMUCH cohort was obtained from clinical testing, transcriptomic data were lacking. Thus, our TCGA cohort findings could not be validated. Instead, we performed immunohistochemical staining analysis of pathological sections to validate the immune activation status of the Epigenetic _Mut group, but a larger-scale validation remains necessary. Furthermore, the application of ICIs in MSS-CRC has not been standardized, and most patients enrolled in our study were patients who had experienced multiple failed lines of treatment, bringing considerable heterogeneity to the population of this study. Therefore, future prospective studies with larger cohort studies are needed.

## Conclusion

In conclusion, our data suggest that identifying epigenetic-related gene mutations might help select the right immunotherapy for MSS-CRC patients and can be used as a biomarker to predict ICI therapy effectiveness. Importantly, the status of epigenetic-related gene mutations is highly accessible from clinical genetic testing, although it is often overlooked by clinicians. Further exploration of the molecular mechanisms underlying the increased effectiveness in specific MSS-CRC patients and prospective clinical trials are therefore warranted.

## Data availability statement

The data presented in the study are deposited in the Genome Sequence Archive in National Genomics Data Center repository (https://ngdc.cncb.ac.cn/gsa-human), accession number GSA-Human: HRA003408.

## Ethics statement

The study was approved by the Ethics Committee of Harbin Medical University Cancer Hospital (Harbin, China). Informed consents were obtained from all patients before surgery and during the experimental procedures.

## Author contributions

CL: Data curation, Formal analysis, Funding acquisition, Investigation, Methodology, Project administration, Software, Validation, Visualization, Writing - original draft, Writing - review & editing. HX: Data curation, Formal analysis, Investigation, Methodology, Software, Validation, Visualization, Writing - original draft, Writing - review & editing. LC: Investigation, Methodology, Validation, Writing - original draft, Writing - review & editing. LF: Investigation, Methodology, Writing - review & editing. SH: Investigation, Writing - review & editing. YR: Investigation, Writing - review & editing. WZ: Conceptualization, Project administration, Resources, Supervision, Writing - review & editing. YZ: Conceptualization, Funding acquisition, Project administration, Resources, Supervision, Writing - review & editing. The work reported in the paper has been performed by the authors, unless clearly specified in the text. All authors contributed to the article and approved the submitted version.

## Funding

This work was supported by grants from the National Natural Science Foundation of China (No. 82102858), the Natural Science Funding of Heilongjiang (No. YQ2022H017), nN10 Excellent Discipline Construction Program (No. Hepatobiliary and Pancreatic Tumor 2017); Basic scientific research projects of universities in Heilongjiang Province (No. 2020-KYYWF-1465); Top Young Talents Project of HMUCH (No. BJQN2021-01); Haiyan Research Fund of Harbin Medical University Cancer Hospital (No. JJMS2022-04), and Beijing Science and Technology Innovation Medical Development Foundation (No. KC2021-JX-0186-102, KC2021-JX-0186-69).

## Acknowledgments

The results of the pan-cancer analysis were based on data generated by TCGA Research Network: https://www.cancer.gov/tcga. The authors thank AiMi Academic Services (www.aimieditor.com) for English language editing services.

## Conflict of interest

The authors declare that the research was conducted in the absence of any commercial or financial relationships that could be construed as a potential conflict of interest.

## Publisher’s note

All claims expressed in this article are solely those of the authors and do not necessarily represent those of their affiliated organizations, or those of the publisher, the editors and the reviewers. Any product that may be evaluated in this article, or claim that may be made by its manufacturer, is not guaranteed or endorsed by the publisher.
