# Supplementary figures and images for: Epigenetic-related gene mutations serve as potential biomarkers for immune checkpoint inhibitors in microsatellite-stable colorectal cancer
Source: Front Immunol. 2022 Nov 21;13:1039631. doi: 10.3389/fimmu.2022.1039631 (PMC9720302; doi:10.3389/fimmu.2022.1039631)

**Supplementary Figure 1. Flowchart of the clinical cohort consolidation from H MUCH.**

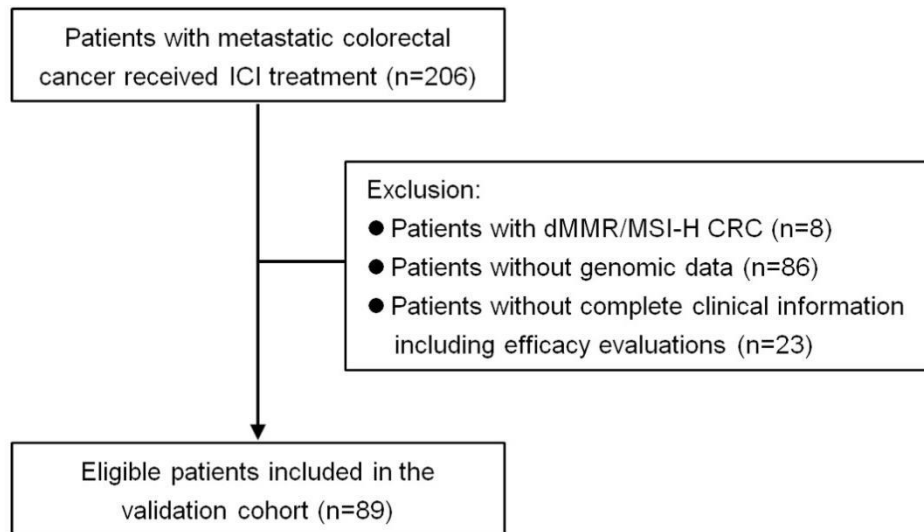

Supplement: Supplementary file 1 [file Image_1.pdf]
