# Supplementary material for: Epigenetic-related gene mutations serve as potential biomarkers for immune checkpoint inhibitors in microsatellite-stable colorectal cancer
Source: Front Immunol. 2022 Nov 21;13:1039631. doi: 10.3389/fimmu.2022.1039631 (PMC9720302; doi:10.3389/fimmu.2022.1039631)
Supplement: Supplementary file 2 [file Table_1.docx]

**Supplementary Table 1. Gene set of epigenetic regulation involved in the current study.**

| **Category** | **Function** | **No.** | **Gene Symbol** |
| --- | --- | --- | --- |
| DNA modifiers | Methylation | 7 | *DNMT1, DNMT3B, DNMT3L, TET1, TET2, TET3, TDG* |
| Histone modifiers | Acetylation | 25 | *EP300, KAT6A, KAT6B, ESA1, dMOF, KAT7, GNAT1, GNAT2, GNAT3, GLYAT, HGSNAT, HDAC1, HDAC2, HDAC4, HDAC7, HDAC8, HDAC9, HDAC10, HDAC11, SIRT1, SIRT2, SIRT3, SIRT5, SIRT6, SIRT7* |
|  | Methylation | 8 | *KMT2A, KMT2B, KMT2C, KMT2D, DOT1L, KDM5A, KDM5B, KDM6B* |
|  | Ubiquitination | 6 | *BRCA1, RING1A, RING1B, BMI1, MYSM1, OTLD1* |
|  | Phosphorylation | 5 | *AURORA, AURORB, MSK1, MSK2, MST1* |
| Chromatin remodelers | SWI/SNF | 5 | *SMARCA4, ARID1A, PBRM1, SMARCB1, ATRX* |
|  | ISWI | 3 | *SMARCA1, SMARCA5, RSF1* |
|  | CHD | 9 | *CHD1, CHD2, CHD3, CHD4, CHD5, CHD6, CHD7, CHD8, CHD9* |
