# Supplementary material for: Epigenetic-related gene mutations serve as potential biomarkers for immune checkpoint inhibitors in microsatellite-stable colorectal cancer
Source: Front Immunol. 2022 Nov 21;13:1039631. doi: 10.3389/fimmu.2022.1039631 (PMC9720302; doi:10.3389/fimmu.2022.1039631)
Supplement: Supplementary file 3 [file Table_2.docx]

**Supplementary Table 2. Gene set of epigenetic regulation in the panel of 825 cancer-related genes of the HMUCH cohort.**

| **Category** | **Function** | **No.** | **Gene symbol** |
| --- | --- | --- | --- |
| DNA modifiers | Methylation | 4 | *DNMT1, DNMT3B, TET1, TET2* |
| Histone modifiers | Acetylation | 7 | *EP300, KAT6A, HDAC1, HDAC2, HDAC4, HDAC8, HDAC9* |
|  | Methylation | 6 | *KMT2A, KMT2B, KMT2C, KMT2D, DOT1L, KDM5A* |
|  | Ubiquitination | 1 | *BRCA1* |
|  | Phosphorylation | 3 | *AURKA, AURKB, MST1* |
| Chromatin remodelers | SWI/SNF | 5 | *SMARCA4, ARID1A, PBRM1, SMARCB1, ATRX* |
|  | ISWI | 1 | *SMARCA1* |
|  | CHD | 3 | *CHD2, CHD4, CHD7* |
