# Supplementary material for: Epigenetic-related gene mutations serve as potential biomarkers for immune checkpoint inhibitors in microsatellite-stable colorectal cancer
Source: Front Immunol. 2022 Nov 21;13:1039631. doi: 10.3389/fimmu.2022.1039631 (PMC9720302; doi:10.3389/fimmu.2022.1039631)
Supplement: Supplementary file 4 [file Table_3.docx]

**Supplementary Table 3. Gene signatures used to analyze the different components of immune responses.**

| **Gene signature** | **Gene symbol** |
| --- | --- |
| IFN-γ pathway | *CD3D, CD3E, CIITA, CD2, HLA-DRA, IL2RG, HLA-E, CXCR6, LAG3, NKG7, GZMK, CCL5, TAGAP, CXCL10, STAT1, GZMB, IDO1* |
| Antigen presentation | *B2M, HLA-A, HLA-B, HLA-C, HLA-DMA, HLA-DMB, HLA-DOA, HLA-DOB, HLA-DPA1, HLA-DRA, HLA-DRB1, HLA-DRB5, NLRC, PSMB8, PSMB9, TAP2* |
| Cytotoxic T-cell function | *ARG1, BTLA, CD160, CD80, CD86, CD8A, GZMA, GZMB, IFNG, KLRG1, NOS2, PRF1, TNFR14* |
| Immune checkpoint | *CD274, CTLA4, HAVCR2, IDO1, IDO2, IL4I1, LAG3, PDCD1, PDCD1LG2, TNFRSF4,* |
| Myeloid-derived suppressor cells (MDSC) | *CD14, CD33, FUT4, ITGAM, TNF* |
| M2 macrophages | *CD163, CD163L1, CD209, F13A1, FOLR2, MRC1, MS4A4A* |
| Treg cells | *FOXP3, ICOS, IL10, IL2RA, TGFB1* |
| Natural Killer cells | *CD56, FCGR3A* |
