# Supplementary material for: Epigenetic-related gene mutations serve as potential biomarkers for immune checkpoint inhibitors in microsatellite-stable colorectal cancer
Source: Front Immunol. 2022 Nov 21;13:1039631. doi: 10.3389/fimmu.2022.1039631 (PMC9720302; doi:10.3389/fimmu.2022.1039631)
Supplement: Supplementary file 5 [file Table_4.docx]

**Supplementary Table 4. Association of epigenetic gene status and clinical outcomes in the validation set of MSS-CRC patients receiving ICI therapy.**

|  | **Particular Epigenetic_Mut***  **(n =16)** | **Epigenetic_Mut**  **(n = 24)** | **Epigenetic_Wt**  **(n = 65)** |
| --- | --- | --- | --- |
| CR (N/%) | 0, 0.00 | 0, 0.00 | 0, 0.00 |
| PR (N/%) | 8, 50.00 | 9, 37.50 | 10, 15.38 |
| SD (N/%) | 5, 31.25 | 7, 29.17 | 14, 21.54 |
| PD (N/%) | 3, 18.75 | 8, 33.33 | 41, 63.08 |
| ORR (N/%) | 8, 50.00 | 9, 37.50 | 10, 15.38 |
| DCR (N/%) | 13, 81.25 | 16, 66.67 | 24, 36.92 |

* Particular Epigenetic_Mut: Include ARID1A, ATRX, KMT2A/B/C/D, TET1-3.

** CR, complete response; PR, partial response; SD, stable disease; PD, progressive disease.
